# A review of asthma care in 50 general practices in Bedfordshire, United Kingdom

**DOI:** 10.1038/s41533-018-0093-7

**Published:** 2018-07-26

**Authors:** Mark L Levy, Fiona Garnett, Adedayo Kuku, Inna Pertsovskaya, Eddie McKnight, John Haughney

**Affiliations:** 1 Respiratory Clinical Lead, Harrow Clinical Commissioning Group, Harrow, UK; 2 NHS Bedfordshire Clinical Commissioning Group, Bedford, UK; 3 NSHI Ltd, Swaffham, Norfolk, UK; 4 Clinical R&D, NHS Greater Glasgow & Clyde, Glasgow, UK

## Abstract

The United Kingdom (UK) National Review of Asthma Deaths (NRAD) (2011–2014) identified a number of contributory risk factors which had not previously been recognized by those caring for people with asthma. Only one of the 19 NRAD recommendations has so far been implemented nationally, and that only partially, and as yet systems are not in place to identify patients at risk of attacks and dying from asthma. In 2015/2016 Bedfordshire Clinical Commissioning Group (CCG) in England, UK, initiated a quality asthma audit of people with asthma to identify some of the risk factors identified in the NRAD report with the aim of optimizing patient care. Fifty (89%) of the General Practices caring for 415,152 patients (27,587 diagnosed with asthma (prevalence 7%; range 4–12%)), participated and the results identified a wide variation in process of care and presence of risk factors including: excess short acting reliever and insufficient preventer prescriptions, failure to issue personal asthma action plans, and to perform annual reviews or check inhaler technique. Identification of these patients involved high-intensity input by trained asthma nurses using sophisticated data extraction software. GP computer systems used in primary care currently do not have the functionally, without the need for manual audit, to implement the NRAD recommendations, starting with the identification of patients at risk. Modifications to existing systems within both primary and secondary care are required in order to prevent unnecessary deaths related to asthma. There is a pressing need to move towards a more pro-active model of care.

## Asthma: Encouraging proactive care

Pro-active disease management for patients with asthma is crucial and could be vastly improved with an overhaul of computer systems in primary care. Following the UK National Review of Asthma Deaths (NRAD) in 2011–2014, multiple mortality risk factors were identified that have yet to be factored into asthma care. The NHS Bedfordshire Clinical Commissioning Group, with support from NSHI and Dr Mark L Levy (Harrow CCG), conducted a manual audit of 50 Bedfordshire doctors’ surgeries to identify relevant NRAD recommendations to optimize patient care. Levy’s team found marked variations in asthma care between the surgeries. Risk factors present included excessive short-acting reliever inhaler prescriptions and inadequate prescribing of preventer inhalers, together with limited implementation of personal asthma plans and annual reviews. The researchers highlight the limitations of existing computer systems for these purposes and recommend an overhaul of asthma tracking and automated alerts.

## Introduction

In 2014 the Royal College of Physicians in London produced a report *Why asthma still kills* following the National Review of Asthma Deaths (NRAD)^1^

This report was the result of a confidential enquiry into the deaths of 276 people in the UK which both highlighted some worrying failures of the care of those people with confirmed asthma death (195) and confirmed previous publication findings that most asthma deaths are associated with major preventable factors.

The key findings of the NRAD included:^1^

- Failure to recognize risk (e.g., poor current symptom control, previous attacks, trigger factors, risk factors for asthma deaths known at the time)^2^
- Excess prescriptions of Short Acting beta-agonist bronchodilators (SABAs)—39% had more than 12 SABA prescribed in the year before death
- Insufficient prescription of Inhaled corticosteroid (ICS) preventer inhalers—38% less than 4 ICS inhalers in the year before death
- Failure to follow patients up after attacks
- Failure to adhere to the UK asthma guidelines^2^

The NRAD report included 19 recommendations that clinicians responsible for the care of people with asthma should observe in order to reduce the number of preventable asthma attacks, improve care, end any complacency regarding the management of this chronic disease, and highlight basic minimum standards that must be met. In order for the NRAD recommendations to be implemented, primary care and other clinicians must be aware of known risk factors for future asthma attacks (i.e., Table 11 in SIGN/BTS,^2^ chapter 2, Box 2-2, in the Global Initiative for Asthma (GINA) strategy document),^3^ and the NRAD report entitled “Why Asthma Still Kills”^1,4^ must have access to systems that are not resource-intensive in order to enable the pro-active identification of “at risk” patients with optimization of their care.

In 2015/2016 Bedfordshire Clinical Commissioning Group (CCG) initiated a quality asthma audit and patient review as part of their Prescribing Incentive Scheme. An aim was to clinically audit the population of people with asthma to identify some of the risk factors identified in the NRAD report and therefore to identify proactively “high risk asthma patients”. Once identified, these high risk patients, could be prioritized for review so that their asthma management could be optimized, targeting resources at the point of greatest need. This report describes the immediate successes of this project and highlights areas where insufficient processes exist in currently held routine NHS data to allow an accurate audit of NRAD recommendations without the need for a significant level of manual audit. This thoughtful and useful audit reported here is one of the first of its kind in the UK and the results are believed to be generalizable across the NHS in England.

## Results

Fifty of the 56 (89%) Bedfordshire practices participated by providing access to their computerized data, and 13 (25%) agreed to a face-to-face review of their asthma patients by NSHI respiratory specialist nurses.

**Fig. 1:**
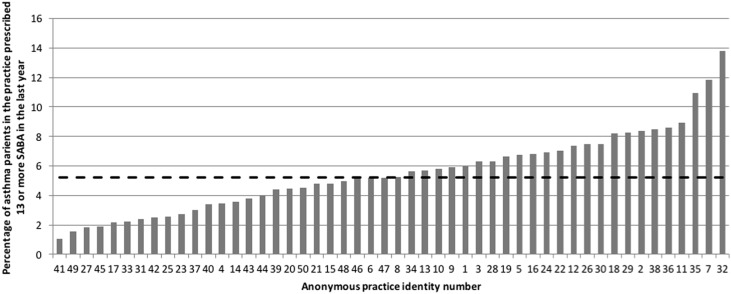
Patients prescribed >12 Short Acting Beta-2 Bronchodilator (SABA) inhalers in previous year as % of total asthma population (range 1–14%, median 5.2%, (hashed line))

**Fig. 2:**
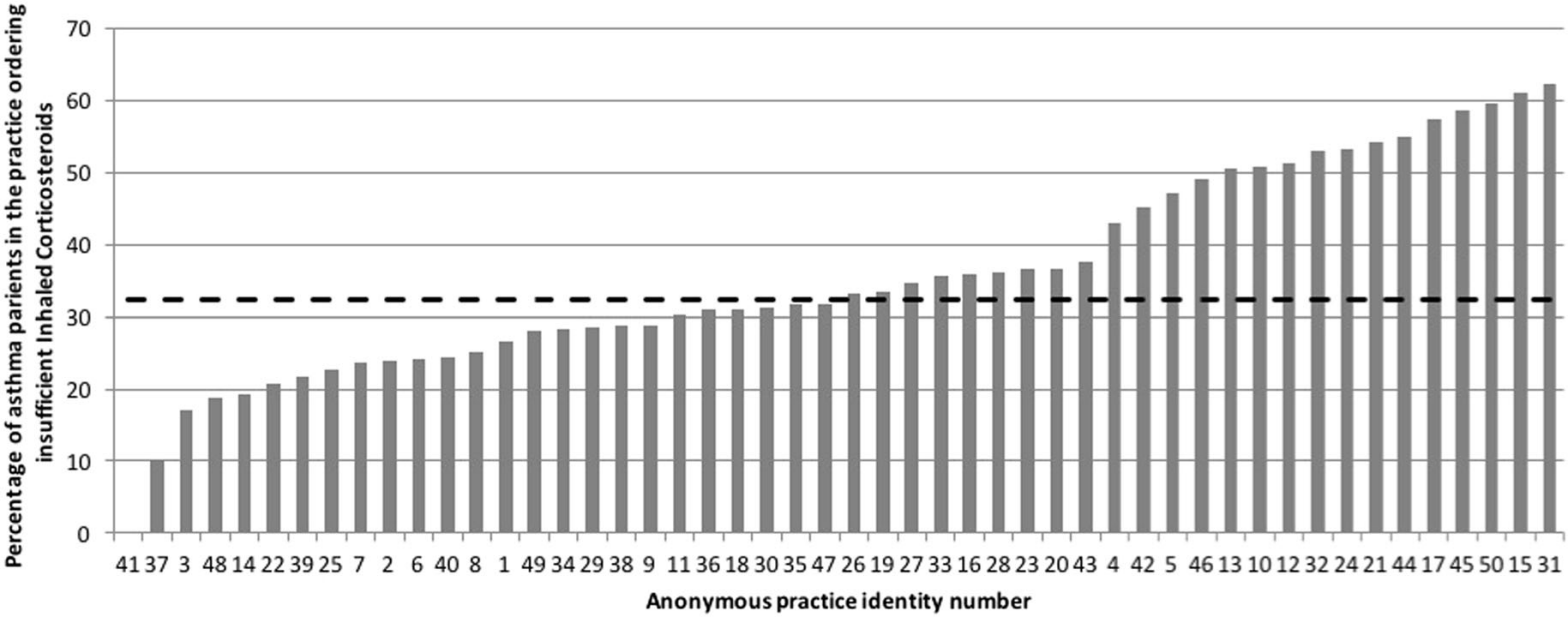
Percentage of patients in practices collecting insufficient (<75%) authorized inhaled corticosteroid (ICS) prescriptions (range 10–62%, median 32% (hashed line))

**Fig. 3:**
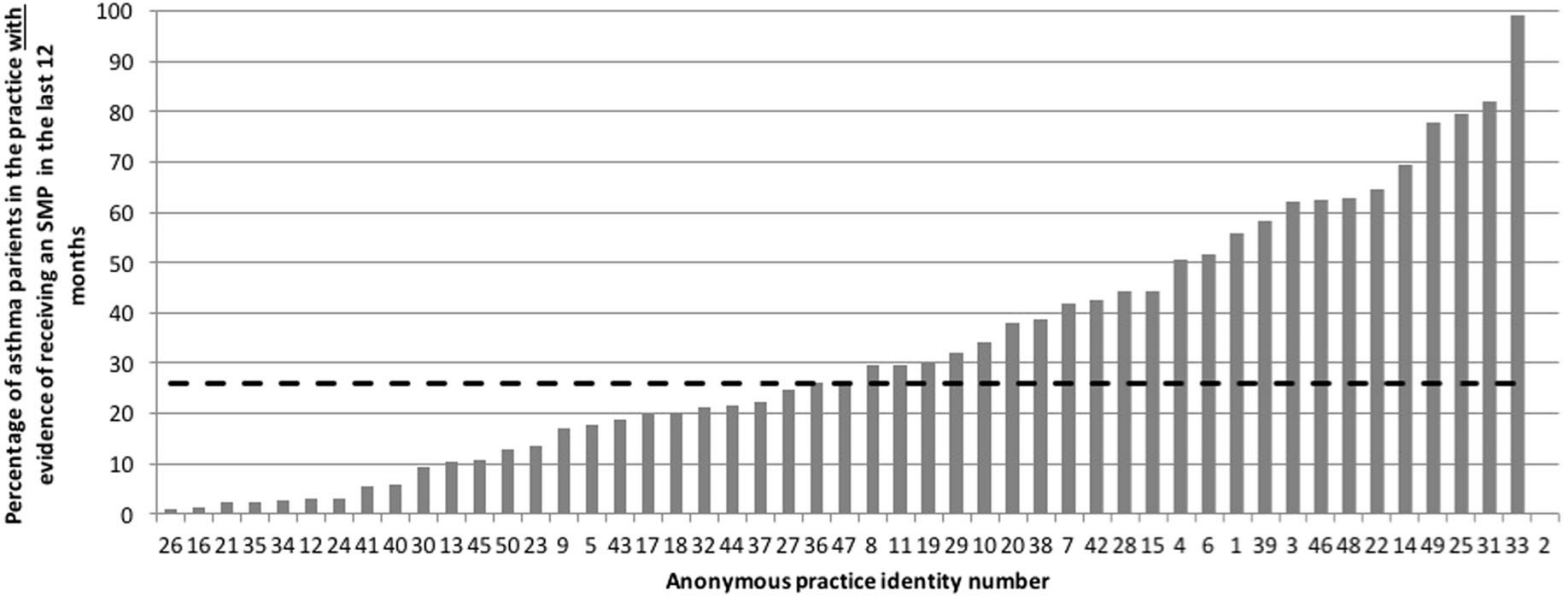
Number of practices with evidence in the medical records of provision of PAAP as % of total asthma population in the practice (range 0.6–98% & median 26% (hashed line))

**Fig. 4:**
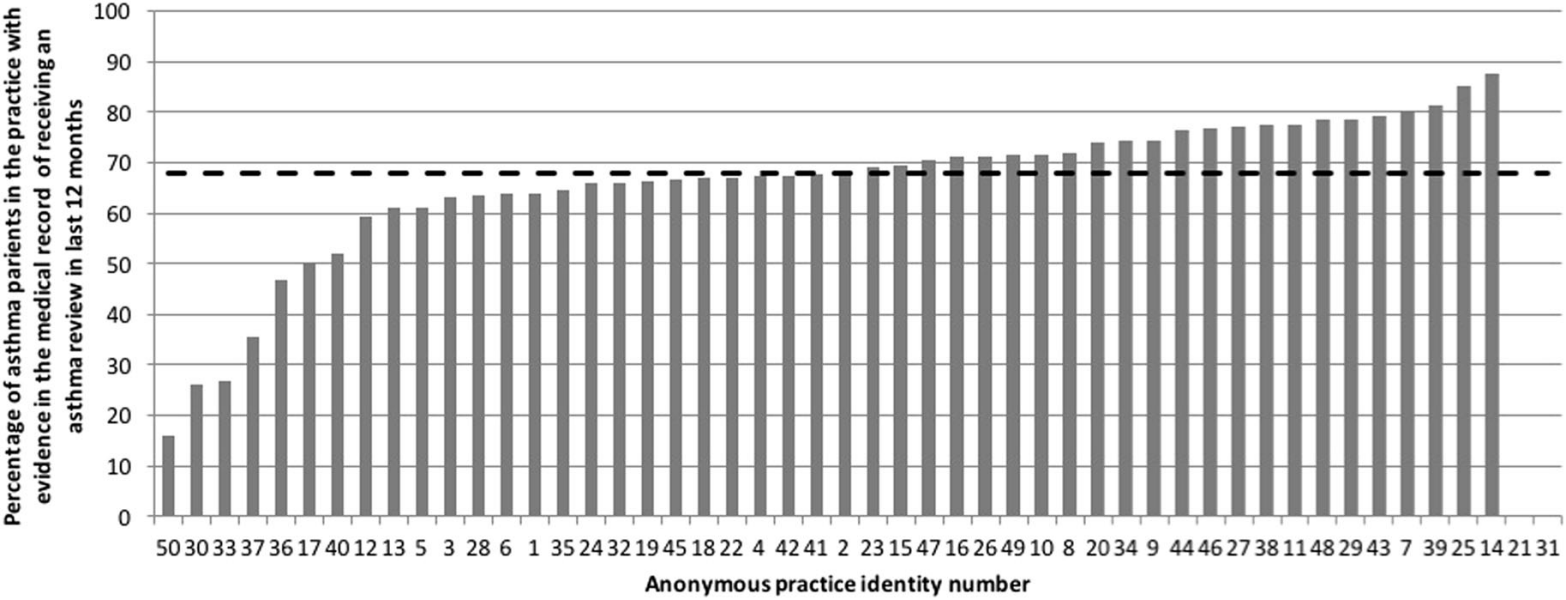
Number with evidence of a record of an annual review as % of total asthma population in the practices (range 15.8–87.4% & median 68% (hashed line)

**Fig. 5:**
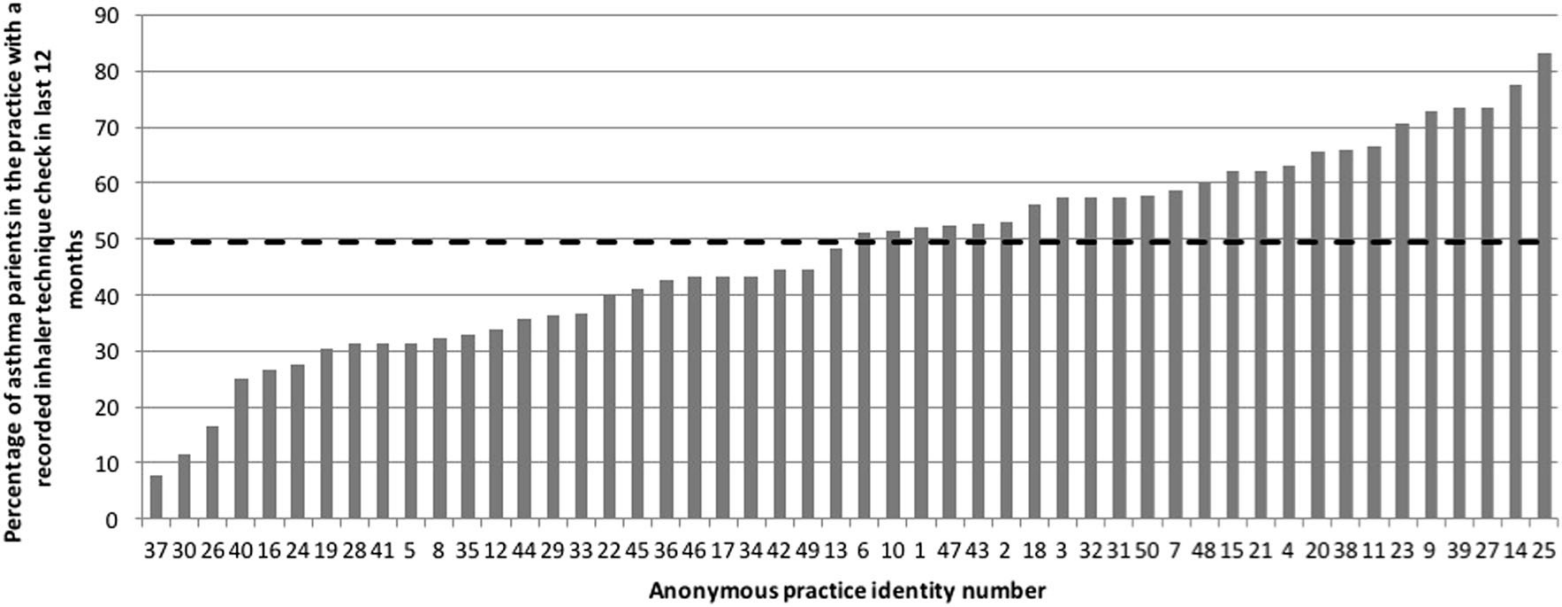
Numbers of patients who had evidence that their inhaler technique was checked in the last year as % of total asthma population (range 7.5–83.1%, Median 49.6% (hashed line))

The anonymised results from the 50 practices are shown at individual practice level in the figures below, and anonymized data for five of the risk factors identified in the NRAD report are shown in Figs. 1–5.

**Fig. 6:**
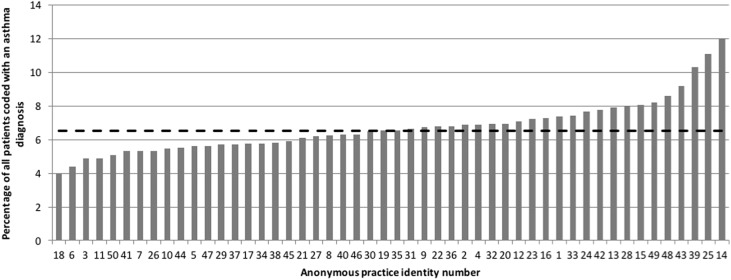
Prevalence (%) of total practice population diagnosed (Read Coded) with asthma (range 4–12%, median (hashed line) 6.5%)

The total number of patients in the 50 participating practices was 415,152 of whom 27,587 (7%) had a Read Code relating to a diagnosis of asthma. The mean number of patients (all ages) in the 50 practices was 8303 (range 2000–17,100), with an asthma population (by Read code) mean of 552 (range 126–1415), 7% (range 4–12%). There was considerable variation in the prevalence of diagnosed asthma in the practices (Fig. 6).

Excess SABA relievers were prescribed in all of the practices. In half of the 50 practices more than 5% (1 in 20) of the patients with asthma were prescribed more than 12 SABA inhalers in the previous year (Fig. 1).

Some patients in all of the practices were not collecting sufficient preventer inhalers required to control their asthma in the previous year (Fig. 2).

Figure 3 shows the distribution of the percentage of asthma patients per practice with had evidence in their records of provision of personal asthma action plans (PAAPs) to help them understand their medication, how to recognize danger and what to do when this occurs (Fig. 3).

Over two thirds of patients in the practices had received an annual review of their asthma (Fig. 4).

Substantial unsuccessful attempts to collect healthcare utilization in hospital (admissions and A&E attendance) were made, involving hand searches of patient records. It was not possible to extract these data from the GP computer systems. Although appropriate coding of correspondence received from hospital would make this possible, Similarly, for the same reasons, we were unable to assess the proportions of patients reviewed following their attacks.

## Discussion

This useful audit, promoted and conducted by the Bedfordshire CCG, highlights a number of important issues.

### Main findings

Variation in clinical processes and outcomes in the UK is well known, and the subject of considerable attention in NHS England. The Right Care program (http://bit.ly/2BurzNE) utilizes national data to compare similar CCGs and identify areas for improvement. (http://bit.ly/2BgFDpG) In this audit, there was marked variation between practices in meeting the standards set. According to the medical records, in this CCG, diagnosed asthma prevalence ranged from 4 to 12%. Explanations for this variation include differences in approaches to diagnosis of asthma, coding, or medical practice variation.

The NRAD highlighted the failure to recognize risk of attacks and death due to excess SABA usage (an issue which was identified 20 years earlier)^5,6^ as a major factor in those who died from asthma attacks. It is therefore of concern that a high proportion of asthma patients are still prescribed excess reliever inhalers. Asthma guidelines state that more than four puffs of SABA a week constitutes poor current asthma symptom control;^2,3^ therefore, a person with well-controlled asthma should need to use less than two inhaler canisters of SABA a year, and many of the patients depicted in Fig. 1 could well have been suffering from poor current symptom control. A recent study reported that the prescription of more than three salbutamol inhalers a year is associated with increased hospital admissions.^7^ While some patients may have been prescribed spare salbutamol reliever inhalers, the key message is that those prescribed more should be urgently contacted and if more than four puffs of the drug is being used weekly, an urgent asthma optimization review is needed.

Across the practices in our audit, a median of 32% of patients ordered <75% of the authorised prescriptions for ICS. Failure to collect ICS prescriptions was also highlighted as a major preventable factor in the deaths of many of those studied in the NRAD. The link between more use of ICS and reduction of asthma deaths has also been known for many years,^8^ as has increased risk of hospital admission been associated with underuse of inhaled corticosteroids.^7^ Furthermore, in the case of children and young people, failure by parents to collect medication may signal a safeguarding issue.

Therefore, practices should strive, ideally, to identify patients who are taking more than four puffs of SABA a week (prescribed more than three reliever inhalers a year) and certainly more than six inhalers a year, or collecting less than 80% of their required ICS; either through practice prescribing systems checks or by involving community pharmacists many of whom now have quality indicators in this area. It might be that SABAs should not be included in repeat prescribing authorization (with a proviso for emergency situations with appropriate prompt for clinical review. A further possible action for practitioners could include closer scrutiny of prescribed medication with recall of patients prescribed more than six SABA inhalers pro-rata in the previous year; and those who are collecting insufficient preventer medication.

This audit demonstrated that the gathering of information regarding (inappropriate) prescribing patterns in people with asthma was not straightforward. Extraction tools for different UK computer systems require unique design. In general, while collecting numbers of prescriptions is achievable, identifying numbers of inhaler devices prescribed, is not. This is an issue that could and should be addressed by medical software companies, together with systems for identification of “at risk” patients prescribed excess SABA as recommended in the NRAD.

Patients who are unable to use their inhaler device correctly may receive less, or often none, of their prescribed medication. In this audit, less than half of the patients had evidence of their inhaler technique being checked. Yet, over 60% had evidence of an asthma review being done; an asthma review should be performed at least once a year, after attacks and when treatment is changed, should include assessment of inhaler technique, and should be performed each time medication is changed. This could also be explained by the variable coding and recording of an inhaler technique assessment noted in this audit.

Since 1992 the British asthma guideline,^2^ and latterly the NICE Quality Statement 25^9^ as well as NRAD^1^ has stated that patients should be reviewed within two working days of treatment for an asthma attack. The post attack review is intended to determine (i) whether the attack has resolved, and therefore whether additional treatment or referral is warranted; (ii) the reasons for the attack (which may be due to inadequate medication or collection thereof, poor inhaler technique, exposure to triggers or adverse drug effects such as beta blocker medication, or other causes); and (iii) to optimize therapy including correcting inhaler technique or providing a different type of inhaler, and modification or provision of a personalized asthma action plan). Unfortunately we were unable to ascertain in this audit, whether patients had been treated for attacks or whether they were reviewed post attack. An action following this audit could be to ensure that the computer record is coded appropriately (e.g., READ Code H333 for those treated for acute asthma exacerbations, irrespective of where this occurred). Better still, linking electronic hospital discharge, emergency room, and out-of-hours data with the primary care patient record would support a seamless transfer of patient care from secondary to primary care. “Red flag” systems currently being developed, for example, in North West London, could highlight patients with risk of exacerbations of asthma in need of treatment optimization reviews, thus satisfying one of the key NRAD recommendations and thus help to avoid unwanted outcomes.

A patient who has been provided with a PAAP is four times less likely to have an asthma attack than someone who has not.^10^ In this audit, only one practice had provided nearly all of their patients with a plan; the wide range in provision from 0 to 98% is clearly an issue to address. A number of examples of PAAPs are available^11,12^ A simple plan that could be implemented immediately could be incorporated within prescriptions for short acting relievers; i.e., the instruction could read: “take one or two puffs for cough, wheeze or shortness of breath, and if this doesn’t help or relief doesn’t last 4 h, contact your doctor or asthma nurse urgently”.

### Strengths and limitations of this study: implications for future research, policy, and practice

The activity within Bedfordshire took ~150 nurse audit days to identify the “at risk” groups across the 50 participating practices. While this process is crucial in order to improve outcomes for patients with asthma, it is not a feasible model without external support to facilitate due to the pressures on resources within primary care. It was extremely difficult for primary care staff to identify patients who had suffered from attacks treated either in practice or in hospital. Electronic data recording was incomplete. Modifications to existing systems within both primary and secondary care are required in order to prevent unnecessary attacks and deaths related to asthma. This is particularly pertinent as many of the high risk patients may by definition need to be identified by computer systems as they may not attend the practice for routine review. In instances of vital patient management, every support should be provided to busy clinicians to assist this activity. Ideally, internal prompts could be made available, and data linkage across healthcare information technology systems should become a priority. As part of the development cycle, Bedfordshire CCG has now developed an asthma review template which includes NRAD recommendations alongside guideline advice.

The NRAD provided evidence that over 60% of asthma deaths are potentially preventable; data over the last 50 years indicates that up to 90% of these deaths are preventable. The NRAD report made 19 recommendations for improving asthma care; and only one of these has been partially implemented nationally, i.e., a National Audit of acute asthma management in secondary care is being set up; current problems with accessing high-quality General Practice data are being addressed (personal communication). Therefore it is up to individual commissioning groups and clinicians to implement the rest of the NRAD recommendations; starting with:

1. The asthma community and/or CCGs should use the outputs from NRAD, in conjunction with audits such as this to,

- Agree a consistent approach to diagnosis of asthma, perhaps incorporating expertise from asthma specialists.
- Delegate asthma reviews only to appropriately trained individuals and these should be performed at least once a year and after every attack—because asthma is a chronic ongoing disease.
- Agree a system for ongoing identification of patients at risk of attacks, and for optimizing their care. This must be a dynamic process because someone who is not on a “risk register” whose risk status changes, should be identified as such with urgent optimization of care.
  - For example, identify and review patients who have collected more than six salbutamol inhalers in a year (or pro-rata)^10^
  - Refer all patients who have had 2 or more asthma attacks in the last year to a specialist (in primary or secondary care)^1^
  - In the UK, attach, preferably automatically, READ Codes for all patients who have had an asthma attack (hospital or primary care) with an appropriate code (H333.) and ensure a post attack review is conducted by an appropriately trained clinician, ideally within 2 working days, or at least before they run out-of-oral corticosteroids to optimize their care^3,9^
- Work to correct deficiencies in systems to identify other parameters that indicate at risk asthma, thus allowing further routine identification of those at risk, to allow appropriate action (locus of care, new methods for assessing compliance)

- Healthcare providers should work to link data relating to patients’ attendance at health providers with asthma events (primary care, out-of-hours, drop in clinics, A&E, secondary care admissions) to ensure an overall picture of the whole patient experience is captured, and addressed. Furthermore, where a patient is deemed to have severe, or difficult to treat asthma, designate one key clinician to provide continuity of care for the patient and their family.
- Designate a named asthma clinical lead in each practice

## Conclusions

This audit has demonstrated, within one CCG, the feasibility of identifying patients at risk of preventable asthma attacks in 50 general practices caring for nearly 30,000 people with asthma, and the considerable variation in care of patients. Identification of these patients involved high-intensity input by trained asthma nurses using sophisticated data extraction software. GP computer systems used in primary care, currently do not have the functionality, without the need for manual audit, to implement the NRAD recommendations starting with the identification of patients at risk. Modifications to existing systems within both primary and secondary care are required in order to prevent unnecessary deaths related to asthma. There is a pressing need to move towards a more pro-active model of care.

## Methods

All general practices in Bedfordshire were invited to participate in the audit. Data for the preceding 12 months was extracted on a rolling basis from each of the practices starting from 22 September 2015 until the 7th April 2016. Registered patients with a Read Coded diagnosis of asthma (H33.) were identified by an electronic search of patient records. This was conducted by a research nurse utilizing a bespoke MIQUEST data extraction tool at each general practice participating in the audit. In the case of inhalers prescribed, the extracted data included details of inhaled medications but not the number of inhalers of each type prescribed. Therefore, in those practices with a policy to prescribe more than one inhaler item each time (say for a two or three month supply of medication), a respiratory specialist nurse did a manual assessment of the prescriptions to determine exactly how many items (i.e., inhalers) were prescribed for each patient during the 12 months of the audit. Daily defined doses were identified and extracted where prescribing instructions on the number of doses per day were detailed. Algorithms were created to use this prescribing data to calculate individual patient’s annual compliance with prescriptions for inhaled preventer and reliever medication. Non-compliance with “controller” medication was defined as 75% or less of dose, per prescriptions collected versus annual requirement according to prescribed doseage.

Substantial unsuccessful attempts to collect healthcare utilization in hospital (admissions and A&E attendance) were made, involving hand searches of patient records. It was not possible to extract these data from the GP computer systems. Although appropriate coding of correspondence received from hospital would make this possible, Similarly, for the same reasons, we were unable to assess the proportions of patients reviewed following their attacks.

The data was held centrally, in an annonymised non-identifiable format, at a commercial database management company. Data was not normally distributed and therefore Medians were used in describing this. Analysis was performed by IP using Excel and Excel Toolpak.

As this is an annonymised medical audit of records, ethics approval and subject permission was not required.

The standards for the audit were as shown below:

- Identified excess Short Acting Beta-agonist bronchodilator (SABA) reliever usage (defined as >12 inhalers per year)
- Failure by patients to collect authorized repeat prescriptions for Inhaled Corticosteroids (ICS) (defined as collection of <75% of required inhalers per year at the prescribed dose)
- Follow up after hospital discharge for asthma-related admission (defined in this review as within 28 days post discharge)
- Provision of written Personalized Asthma Action Plans (everyone with asthma should have one)^2,9^
- Annual reviews for all patients diagnosed with asthma who are prescribed regular medication
- Inhaler technique routinely checked and recorded in patient notes
- Adherence to guidelines in the practice on the stepping down of patients on an inappropriately high dose of ICS or conversely stepping up patients who required increased medication

In this paper, we present the average results by practice of the clinical audit of the practice records against the set standards detailed above. We were unable at this time to present patient level data, nor were we able to correlate these results with healthcare utilization by patients in these practices.

### Data availability

The data for this paper are not available because the authors do not wish to identify individual practices.
